# Oral health-related quality of life, probable depression and probable anxiety: evidence from a representative survey in Germany

**DOI:** 10.1186/s12903-022-02047-y

**Published:** 2022-01-16

**Authors:** André Hajek, Hans-Helmut König

**Affiliations:** Department of Health Economics and Health Services Research, Hamburg Center for Health Economics, University Medical Center Hamburg-Eppendorf, 20246 Hamburg, Germany

**Keywords:** Oral health, Oral health-related quality of life, Depression, Anxiety, Patient Health Questionnaire (PHQ), Generalized Anxiety Disorder

## Abstract

**Background:**

There is limited knowledge regarding the association between oral health-related quality of life and probable depression and anxiety. Our objective was to examine the association between oral health-related quality of life and probable depression and anxiety in the German population (stratified by sex).

**Methods:**

In sum, n = 3,075 individuals took part in a nationally representative survey (August/September 2021). The well-established Patient Health Questionnaire-9 (PHQ-9; cut-off of 10) and the Generalized Anxiety Disorder-7 (GAD-7; cut-off of 10) were used to quantify probable depression and anxiety, respectively.

**Results:**

The likelihood of probable depression was significantly associated with lower oral health-related quality of life in the total sample (OR: 1.21, 95% CI: 1.18–1.25) and in both women and men. Additionally, the likelihood of probable anxiety was significantly associated with lower oral health-related quality of life in the total sample (OR: 1.17, 95% CI: 1.14–1.21) and in both women and men.

**Conclusions:**

Our study emphasized the association between lower oral health-related quality of life and probable depression as well as anxiety in the general adult population in Germany. Future longitudinal studies are required to confirm our findings.

**Supplementary Information:**

The online version contains supplementary material available at 10.1186/s12903-022-02047-y.

## Background

The prevalence of both depression and anxiety is high in adulthood [1, 2]. Moreover, both are, among other things, associated with labor force participation [3], substance use [4] and even high healthcare costs [5].

There is considerable knowledge regarding the factors that contribute to depression and anxiety in adulthood, for example: socioeconomic factors, lifestyle-related factors, or health-related factors [6, 7].

Moreover, some studies exist examining the association between oral health-related quality of life and probable depression (e.g., [8–16]). They mainly showed an association between lower oral health-related quality of life and a higher likelihood of probable depression [8–10, 13, 15, 16]. Moreover, only two studies also exist investigating the association between oral health-related quality of life and probable anxiety in adulthood. However, these two studies did not explicitly differentiate between anxiety and depression. Both studies showed a significant association between anxiety/depressive symptoms and lower oral health-related quality of life [17, 18].

In sum, there is limited knowledge regarding the association between oral health-related quality of life and probable depression and anxiety in the general adult population in Germany. Therefore, our aim was to close this gap in knowledge. This knowledge is important to identify individuals at risk for depression or anxiety. Moreover, this knowledge is important because probable depression and anxiety can contribute to morbidity and mortality.

## Methods

### Sample

For this study, we used data (n = 3,075 individuals) from a nationally representative online survey (from late August to early September 2021) including individuals 18 to 70 years and residing in Germany (i.e., excluding individuals not residing in Germany; excluding individuals < 18 years as well as excluding individuals > 70 years). Various recruitment sources were used for this online sample such as search engine marketing or opinion platforms. The questionnaire was solely available in one language (German language). Therefore, we strongly assume that individuals with greater difficulties in reading the German language did not participate.

The market research company Respondi recruited the participants from an online panel in a way that it matches the distribution of sex, age bracket as well as the federal state in the German adult population (quotas were from the best4planning 2020). Using these socio-demographic data, a random sample from the population of the online access panel was drawn. Approximately 14,000 individuals were contacted to participate in this study. Two reminders (maximum) were send after the first invitation (with a minimum time interval between two reminders of two days). Digital fingerprint solutions were used to avoid duplicates).

### Outcomes

To measure probable depression, the Patient Health Questionnaire-9 (PHQ-9) was used, consisting of nine items [19]. It is a well-known and valid screening tool. A sum score was calculated. This score ranges from 0 to 27 (higher values reflect more depressive symptoms). The sensitivity was 0.88 and the specificity was 0.88 for major depressive disorder (using a PHQ-9 score of ten or more) [19]. Cronbach’s alpha equaled 0.91 in our study. In accordance with previous recommendations, we also used a PHQ-9 of ten or higher as cut-off [20].

To assess probable anxiety, the Generalized Anxiety Disorder-7 (GAD-7) was used [21], consisting of seven items. A sum score was computed (0–21; higher scores reflect more anxiety symptoms). Based on a GAD-7 score of ten or higher, the specificity was 0.82 and the sensitivity was 0.89. Cronbach’s alpha equaled 0.92 in our study. As common, a cut-off of ten or higher was used [21].

### Independent variables

The key independent variable was oral health-related quality of life. The well-established Oral Health Impact Profile (OHIP-G5) was used to assess it (five original scales: functional limitation; physical disability; physical pain; psychological discomfort; social disability). It encompasses four dimensions: (i) oral function, (ii) orofacial pain, (iii) appearance, and (iv) psychosocial impact. Favorable psychometric characteristics have been shown [22]. The OHIP-G5 ranges from 0 to 20, whereby higher scores reflect *lower* oral health-related quality of life. Cronbach’s alpha was 0.85 in our current study.

As covariates, these factors were included in regression analysis: sex, age, family status (married, living together with spouse; married, not living together with spouse; single; widowed; divorced), highest educational degree (upper secondary school; qualification for applied upper secondary school; polytechnic Secondary School; intermediate Secondary School; Lower Secondary School; currently in school training/education; without school-leaving qualification), and occupational status (full-time employed; retired; other). Moreover, it was adjusted for: alcohol intake (daily; several times per week; once a week; 1–3 times per month; less often; never), smoking status (yes, daily; yes, sometimes; no, not anymore; never smoker), sports activities (no sports activity; less than one hour a week; regularly, 1–2 h a week; regularly, 2–4 h a week; regularly, more than 4 h a week), vaccination against Covid-19 (no; yes), at least having one chronic condition (no; yes) and self-rated health (single item: from 1 = very bad to 5 = very good).

Covariates were selected based on former research (e.g., [8–10, 13, 15, 16]) and theoretical considerations. More precisely, sociodemographic factors may be associated with both lower oral health-related quality of life and depression and anxiety. For example, higher educational level may be associated with higher oral health-related quality of life [23] and a lower likelihood of depression and anxiety [24]. Another example: Being married may also be associated with higher oral health-related quality of life [25] and a lower likelihood of depression and anxiety [26]. Moreover, previous research demonstrated an association between adverse lifestyle factors (no sports activity, currently smoking and high alcohol intake) and lower oral health-related quality of life [27] and a higher likelihood of depression and anxiety [28, 29]. Additionally, having chronic conditions and lower self-rated health have both been shown to be associated with lower oral health-related quality of life [30] and a higher likelihood of depression and anxiety [31]. Vaccination against Covid-19 was used in regression analysis because it can contribute to a lower likelihood of depression and anxiety [32]. Furthermore, we assume that the vaccination status can affect oral health-related quality of life because individuals not being vaccinated against Covid-19 may avoid using dental services due to the fear of being infected [33].

### Statistical analysis

First, key sample characteristics are shown. Thereafter, effect sizes were calculated (total sample and stratified by sex). Finally, multiple logistic regressions were estimated—total sample and also stratified by sex (first: with probable depression as outcome measure; second: second: with probable anxiety as outcome measure). Statistical significance was defined as p value of 0.05 or smaller. Stata 16.1 (Stata Corp., College Station, Texas) was used to perform statistical analyses.

It was checked for multicollinearity. However, the highest variance inflation factors was 3.92 (mean variance inflation factor was 1.76) for the total sample. Therefore, it was concluded that multicollinearity is not a threat for our analysis. Furthermore, some important model diagnostics are shown in Additional file 1: Table S1 and S2.

## Results

### Sample characteristics and effect sizes

Mean age in our sample was 44.5 years, SD: 14.8 years. The age ranges from 18 to 70 years (51.1% were female). Average OHIP-G5 score was 2.2 (SD: 3.3), ranging from 0 to 20. In sum, 50.0% had a OHIP-G5 score of zero. The average PHQ-9 score was 5.7 (SD: 5.5), ranging from 0 to 27. According to the PHQ-9 score of ten or higher, 20.0% of the respondents had probable depression. The average GAD-7 score was 4.1 (SD: 4.7), ranging from 0 to 21. According to the GAD-7 score of ten or higher, 13.4% of the respondents had probable anxiety. Further details regarding the sample characteristics are given in Additional file 2: Table S3. Moreover, our sample and the target cohort are compared in Additional file 3: Table S4.

Effect sizes were also calculated for the association between oral health-related quality of life and both outcomes (see Table 1). We calculated Pearson’s r and Cohen’s d (as appropriate). For example, Cohen’s d for the association between oral health-related quality of life (dichotomous) and depressive symptoms was -0.64 in the total sample (with anxiety symptoms, Cohen’s d was − 0.58 in the total sample). With regard to the negative sign (Cohen’s d), this for example means that individuals with a OHIP-G5 score of zero had lower depressive symptoms compared to individuals with a OHIP-G5 score of at least one. Further details are given in Table 1.

**Table 1:** Effect sizes for the association between oral health-related quality of life and both outcomes

	Probable depression (dichotomous)			Probable anxiety (dichotomous)		
	Total	Men	Women	Total	Men	Women
Oral health-related quality of life (continuous): Pearson’s r	.33	.39	.29	.28	.35	.23

	Depressive symptoms (continuous)			Anxiety symptoms (continuous)		
	Total	Men	Women	Total	Men	Women
Oral health-related quality of life (dichotomous, first group: zero; second group: one or higher): Cohen’s d	− .64	− .71	− .57	− .58	− .64	− .52

### Regression analysis

Findings of multiple logistic regressions are shown in Table 2. A one-unit increase in oral health-related quality of life was associated with both outcomes. More precisely, the likelihood of probable depression was significantly associated with lower oral health-related quality of life in the total sample (OR: 1.21, 95% CI: 1.18–1.25) and in both women (OR: 1.19, 95% CI: 1.14–1.25) and men (OR: 1.24, 95% CI: 1.19–1.29). Additionally, the likelihood of probable anxiety was significantly associated with lower oral health-related quality of life in the total sample (OR: 1.17, 95% CI: 1.14–1.21) and in both women (OR: 1.14, 95% CI: 1.09–1.19) and men (OR: 1.21, 95% CI: 1.16–1.26).

**Table 2:** Determinants of probable depression and probable anxiety. Results of multiple logistic regressions

Independent variables	Probable depression—total sample	Probable depression—Women	Probable depression—Men	Probable anxiety—Total sample	Probable anxiety—Women	Probable anxiety—Men
Oral health-related quality of life	1.21***	1.19***	1.24***	1.17***	1.14***	1.21***
	(1.18–1.25)	(1.14–1.25)	(1.19–1.29)	(1.14–1.21)	(1.09–1.19)	(1.16–1.26)
Potential confounders	✓	✓	✓	✓	✓	✓
Observations	3075	1570	1502	3075	1570	1499
Pseudo-R^2^	.28	.28	.28	.25	.22	.29

Odds ratios are displayed; 95% CI in parentheses

***p < 0.001, **p < 0.01, *p < 0.05, +*p* < 0.10

Potential confounders include sex (as appropriate), age, family status, educational level, occupational status, smoking status, alcohol intake, sports activities, vaccinated against Covid-19, presence of chronic diseases and self-rated health

In further multiple logistic regression analysis (sensitivity analysis), oral health-related quality of life was dichotomized (i.e., OHIG-G5 scores of zero; scores of one or higher): The likelihood of probable depression was significantly associated with low oral health-related quality of life in the total sample (OR: 2.91, 95% CI: 2.33–3.63) and in both women (OR: 2.68, 95% CI: 1.99–3.61) and men (OR: 3.49, 95% CI: 2.48–4.92). Additionally, the likelihood of probable anxiety was significantly associated with low oral health-related quality of life in the total sample (OR: 2.11, 95% CI: 1.64–2.70) and in both women (OR: 1.88, 95% CI: 1.35–2.60) and men (OR: 2.63, 95% CI: 1.76–3.94).

In further sensitivity analysis (please see Additional file 4: Tables S5 to S8), the four dimensions were entered separately to the regression model: (1) difficulty chewing foods and less flavor in food (dimension: oral function), (2) painful aching (dimension: orofacial pain), (3) uncomfortable about appearance (dimension: appearance) and (4) difficulty doing your usual jobs (dimension: psychosocial impact). Particularly strong association were revealed between the dimension psychosocial impact and the two outcomes (followed by painful aching, appearance and oral function).

## Discussion

Based on data from the general adult population in Germany, our objective was to examine the association between oral health-related quality of life and probable depression and anxiety in the German population (also stratified by sex). Mostly medium (to medium-large) effect sizes were identified. After adjusting for several covariates, regressions showed an association between oral health-related quality of life and both probable depression and anxiety in the total sample and in both sexes.

In accordance with most of the results from existing studies from other countries [8–10, 13, 15, 16], our study demonstrated an association between oral health-related quality of life and probable depression in the German population. As the first study, our study also demonstrated an association between oral health-related quality of life and probable anxiety. In terms of effect sizes regarding the association between oral health-related quality of life and probable depression, our findings are mostly similar in comparison to previous studies. For example, based on data from the National Health and Nutrition Examination Surveys (NHANES; United States), a previous study revealed a similar association of poor dental health and probable depression (also based on the PHQ-9) (OR: 1.60, 95% CI: 1.08–2.38) [34]. A similar association between these factors (OR: 1.55, 95% CI: 1.05–2.28) were also made by a study among adults in urban areas of Recife (Brazil) [10].

A possible explanation for these associations may be that oral health contributes to overall health-related quality of life and life satisfaction [35, 36] which in turn could contribute to depression [37]. Additionally, factors such as showing teeth with embarrassment or feeling stigmatized because of the teeth may lower general self-esteem which in turn could increase the likelihood of depression or anxiety [35, 36, 38].

In additional analysis, analyses were also conducted for each dimension [(i) oral function, (ii) orofacial pain, (iii) appearance, and (iv) psychosocial impact]. Findings suggested that the association between oral health-related quality of life and probable depression and anxiety is particularly driven by the dimension psychosocial impact. This also supports our assumption that psychosocial factors mentioned above (e.g., self-worth, satisfaction with life or embarrassment) may be of great importance for this association. We think that far more research in this area is required to clarify the underlying mechanisms.

Psychosocial factors such as loneliness or social isolation may also be of importance for the association between oral health-related quality of life and both outcomes of our study. Actually, previous studies repeatedly showed an association between low oral health-related quality of life and high loneliness levels [39–41]. High loneliness and isolation levels are also associated with low mental health [42].

Most of the existing studies used oral health-related quality as independent variable and depression as outcome measure. However, it also appears plausible that depression contributes to low oral health-related quality of life, for example via bad lifestyle habits (e.g., alcohol intake, smoking, junk food and sedentary lifestyle [43–45]). Thus, future research in this area is urgently required.

Some strengths and limitations are worth noting. For our study, data were taken from a representative survey. The key variables (oral health-related quality of life, depression and anxiety) were quantified using widely used and valid tools. However, it should be emphasized that these are screening tools. Thus, future research is needed to confirm our findings. Additionally, our study has a cross-sectional design (with the well-known limitations regarding causality). Longitudinal studies are required in this area. Furthermore, other factors (e.g., quality of a potential prosthesis) should be included in future studies. A limitation is that our survey was only performed in German language. Moreover, we cannot dismiss the possibility that non-respondents differ from respondents (e.g., regarding health status). However, these potential differences could not be computed.

## Conclusions

In conclusion, our study emphasized the association between lower oral health-related quality of life and probable depression and anxiety (medium to medium-large effect size) in the general adult population in Germany. Future longitudinal studies are required to confirm our findings.

## Supplementary Information

**Additional file 1**. Model diagnostics.

**Additional file 2**. Sample characteristics.

**Additional file 3**. Comparison of the target quote and our sample.

**Additional file 4**. Determinants of probable depression and probable anxiety (additional analyses).

## Data Availability

The datasets used and analysed during the current study are available from the corresponding author on reasonable request for all interested researchers.

## Abbreviations

CI: Confidence interval
GAD-7: Generalized Anxiety Disorder-7
OR: Odds ratio
OHIP: Oral health impact profile
PHQ-9: Patient Health Questionnaire
