# Supplementary material for: Oral health-related quality of life, probable depression and probable anxiety: evidence from a representative survey in Germany
Source: BMC Oral Health. 2022 Jan 16;22:9. doi: 10.1186/s12903-022-02047-y (PMC8761375; doi:10.1186/s12903-022-02047-y)
Supplement: Supplementary file 2 — Additional file 2. Sample characteristics. [file 12903_2022_2047_MOESM2_ESM.docx]

Supplementary Table 3. Sample characteristics (first group: without probable depression and without probable anxiety; second group: only probable depression; third group: only probable anxiety; fourth group: probable depression and probable anxiety) (n=3,075)

| Variables | First group (n=2,402) | Second group (n=261) | Third group (n=59) | Fourth group (n=353) |
| --- | --- | --- | --- | --- |
|  |  |  |  |  |
|  | N (%)  / Mean (SD) | N (%)  / Mean (SD) | N (%)  / Mean (SD) | N (%)  / Mean (SD) |
| Sex: N (%) |  |  |  |  |
| Men | 1235 (51.4%) | 103 (39.5%) | 18 (30.5%) | 146 (41.4%) |
| Women | 1166 (48.5%) | 157 (60.2%) | 41 (69.5%) | 206 (58.4%) |
| Diverse | 1 (0.0%) | 1 (0.4%) | 0 (0.0%) | 1 (0.3%) |
| Age: Mean (SD) | 45.9 (14.5) | 41.4 (15.3) | 38.6 (14.3) | 38.5 (14.1) |
| Marital status: N (%) |  |  |  |  |
| Single / Divorced / Widowed / Married, not living together with spouse | 944 (39.3%) | 139 (53.3%) | 29 (49.2%) | 201 (56.9%) |
| Married, living together with spouse | 1458 (60.7%) | 122 (46.7%) | 30 (50.8%) | 152 (43.1%) |
| Highest educational degree: N (%) |  |  |  |  |
| upper secondary school | 1040 (43.3%) | 103 (39.5%) | 35 (59.3%) | 148 (41.9%) |
| qualification for applied upper secondary school | 257 (10.7%) | 33 (12.6%) | 5 (8.5%) | 33 (9.3%) |
| polytechnic Secondary School | 145 (6.0%) | 11 (4.2%) | 0 (0.0%) | 12 (3.4%) |
| intermediate Secondary School | 680 (28.3%) | 89 (34.1%) | 16 (27.1%) | 103 (29.2%) |
| Lower Secondary School | 269 (11.2%) | 23 (8.8%) | 3 (5.1%) | 52 (14.7%) |
| currently in school training/education | 7 (0.3%) | 1 (0.4%) | 0 (0.0%) | 1 (0.3%) |
| without school-leaving qualification | 4 (0.2%) | 1 (0.4%) | 0 (0.0%) | 4 (1.1%) |
| Occupational status: N (%) |  |  |  |  |
| Full-time employed | 1190 (49.5%) | 102 (39.1%) | 31 (52.5%) | 135 (38.2%) |
| Retired | 405 (16.9%) | 46 (17.6%) | 8 (13.6%) | 40 (11.3%) |
| Other | 807 (33.6%) | 113 (43.3%) | 20 (33.9%) | 178 (50.4%) |
| Smoking status: N (%) |  |  |  |  |
| Yes, daily | 549 (22.9%) | 63 (24.1%) | 7 (11.9%) | 97 (27.5%) |
| Yes, sometimes | 174 (7.2%) | 24 (9.2%) | 9 (15.3%) | 44 (12.5%) |
| No, not anymore | 674 (28.1%) | 76 (29.1%) | 17 (28.8%) | 76 (21.5%) |
| Never smoker | 1005 (41.8%) | 98 (37.5%) | 26 (44.1%) | 136 (38.5%) |
| Sports activities: N (%) |  |  |  |  |
| No sports activity | 627 (26.1%) | 82 (31.4%) | 11 (18.6%) | 114 (32.3%) |
| Less than one hour a week | 467 (19.4%) | 70 (26.8%) | 12 (20.3%) | 80 (22.7%) |
| Regularly, 1-2 hours a week | 562 (23.4%) | 61 (23.4%) | 12 (20.3%) | 79 (22.4%) |
| Regularly, 2-4 hours a week | 391 (16.3%) | 25 (9.6%) | 10 (16.9%) | 47 (13.3%) |
| Regularly, more than 4 hours a week | 355 (14.8%) | 23 (8.8%) | 14 (23.7%) | 33 (9.3%) |
| Alcohol intake: N (%) |  |  |  |  |
| Daily | 143 (6.0%) | 18 (6.9%) | 3 (5.1%) | 22 (6.2%) |
| Several times per week | 450 (18.7%) | 40 (15.3%) | 11 (18.6%) | 63 (17.8%) |
| Once a week | 395 (16.4%) | 34 (13.0%) | 11 (18.6%) | 55 (15.6%) |
| 1-3 times per month | 423 (17.6%) | 44 (16.9%) | 6 (10.2%) | 59 (16.7%) |
| Less often | 545 (22.7%) | 72 (27.6%) | 12 (20.3%) | 86 (24.4%) |
| Never | 446 (18.6%) | 53 (20.3%) | 16 (27.1%) | 68 (19.3%) |
| Vaccinated against Covid-19: N (%) |  |  |  |  |
| No | 445 (18.5%) | 50 (19.2%) | 18 (30.5%) | 80 (22.7%) |
| Yes | 1957 (81.5%) | 211 (80.8%) | 41 (69.5%) | 273 (77.3%) |
| Chronic diseases: N (%) |  |  |  |  |
| Absence of chronic diseases | 1478 (61.5%) | 104 (39.8%) | 33 (55.9%) | 150 (42.5%) |
| Presence of at least one chronic disease | 924 (38.5%) | 157 (60.2%) | 26 (44.1%) | 203 (57.5%) |
| Self-rated health (1 = very bad to 5 = very good): Mean (SD) | 3.8 (0.8) | 3.1 (0.9) | 3.4 (0.9) | 2.9 (1.0) |
| Oral health-related quality of life (OHIP-G5; from 0 to 20,  with higher scores indicating lower oral health-related quality of life): Mean (SD) | 1.6 (2.7) | 3.8 (4.0) | 2.6 (3.2) | 4.9 (4.8) |
