# Supplementary material for: Oral health-related quality of life, probable depression and probable anxiety: evidence from a representative survey in Germany
Source: BMC Oral Health. 2022 Jan 16;22:9. doi: 10.1186/s12903-022-02047-y (PMC8761375; doi:10.1186/s12903-022-02047-y)
Supplement: Supplementary file 3 — Additional file 3. Comparison of the target quote and our sample. [file 12903_2022_2047_MOESM3_ESM.docx]

| ***Sex*** | ***Target quote*** | ***Our sample*** |
| --- | --- | --- |
| Men | 50% | 49% |
| Women | 50% | 51% |
| ***Age group*** |  |  |
| 18 – 29 years | 20% | 21% |
| 30 – 39 years | 19% | 19% |
| 40 – 49 years | 19% | 19% |
| 50 – 59 years | 21% | 22% |
| 60 – 70 years | 21% | 19% |
| ***State*** |  |  |
| Baden-Wuerttemberg | 13% | 13% |
| Bavaria | 16% | 16% |
| Berlin | 4% | 4% |
| Brandenburg | 3% | 3% |
| Bremen | 1% | 1% |
| Hamburg | 2% | 2% |
| Hesse | 7% | 7% |
| Mecklenburg-Western Pomerania | 2% | 2% |
| Lower Saxony | 10% | 10% |
| North Rhine-Westphalia | 22% | 22% |
| Rhineland-Palatinate | 5% | 5% |
| Saarland | 1% | 1% |
| Saxony | 5% | 5% |
| Saxony-Anhalt | 3% | 5% |
| Schleswig-Holstein | 3% | 3% |
| Thuringia | 3% | 3% |

Supplementary Table 4. Comparison of the target quote and our sample.
