# Supplementary material for: Oral health-related quality of life, probable depression and probable anxiety: evidence from a representative survey in Germany
Source: BMC Oral Health. 2022 Jan 16;22:9. doi: 10.1186/s12903-022-02047-y (PMC8761375; doi:10.1186/s12903-022-02047-y)
Supplement: Supplementary file 4 — Additional file 4. Determinants of probable depression and probable anxiety (additional analyses). [file 12903_2022_2047_MOESM4_ESM.docx]

Supplementary Table 5. Determinants of probable depression and probable anxiety. Results of multiple logistic regressions

| Independent variables | Probable depression  - Total sample | Probable depression  - Women | Probable depression  - Men | Probable anxiety -  Total sample | Probable anxiety  - Women | Probable anxiety  - Men |
| --- | --- | --- | --- | --- | --- | --- |
|  |  |  |  |  |  |  |
| Oral function: Difficulty chewing foods | 1.32*** | 1.22* | 1.46*** | 1.33*** | 1.44*** | 1.21+ |
|  | (1.17 - 1.49) | (1.02 - 1.45) | (1.22 - 1.74) | (1.16 - 1.52) | (1.20 - 1.74) | (0.98 - 1.49) |
| Oral function: Less flavor in food | 1.65*** | 1.57*** | 1.73*** | 1.46*** | 1.05 | 1.96*** |
|  | (1.43 - 1.90) | (1.27 - 1.95) | (1.42 - 2.12) | (1.25 - 1.70) | (0.83 - 1.33) | (1.56 - 2.47) |
|  |  |  |  |  |  |  |
|  |  |  |  |  |  |  |
| Potential confounders | ✓ | ✓ | ✓ | ✓ | ✓ | ✓ |
|  |  |  |  |  |  |  |
| Observations | 3,075 | 1,570 | 1,502 | 3,075 | 1,570 | 1,499 |
| Pseudo-R² | .26 | .27 | .27 | .23 | .21 | .28 |

Odds ratios are displayed; 95% CI in parentheses; *** p<0.001, ** p<0.01, * p<0.05, + p<0.10; Potential confounders include sex (as appropriate), age, family status, educational level, occupational status, smoking status, alcohol intake, sports activities, vaccinated against Covid-19, presence of chronic diseases and self-rated health

Supplementary Table 6. Determinants of probable depression and probable anxiety. Results of multiple logistic regressions

| Independent variables | Probable depression  - Total sample | Probable depression  - Women | Probable depression  - Men | Probable anxiety -  Total sample | Probable anxiety  - Women | Probable anxiety  - Men |
| --- | --- | --- | --- | --- | --- | --- |
|  |  |  |  |  |  |  |
| Painful aching: Orofacial pain | 1.79*** | 1.69*** | 1.92*** | 1.57*** | 1.44*** | 1.74*** |
|  | (1.61 - 1.99) | (1.45 - 1.97) | (1.65 - 2.24) | (1.40 - 1.76) | (1.23 - 1.68) | (1.47 - 2.05) |
|  |  |  |  |  |  |  |
| Potential confounders | ✓ | ✓ | ✓ | ✓ | ✓ | ✓ |
|  |  |  |  |  |  |  |
| Observations | 3,075 | 1,570 | 1,502 | 3,075 | 1,570 | 1,499 |
| Pseudo-R² | .25 | .27 | .24 | .22 | .25 | .25 |

Odds ratios are displayed; 95% CI in parentheses; *** p<0.001, ** p<0.01, * p<0.05, + p<0.10; Potential confounders include sex (as appropriate), age, family status, educational level, occupational status, smoking status, alcohol intake, sports activities, vaccinated against Covid-19, presence of chronic diseases and self-rated health

Supplementary Table 7. Determinants of probable depression and probable anxiety. Results of multiple logistic regressions

| Independent variables | Probable depression  - Total sample | Probable depression  - Women | Probable depression  - Men | Probable anxiety -  Total sample | Probable anxiety  - Women | Probable anxiety  - Men |
| --- | --- | --- | --- | --- | --- | --- |
|  |  |  |  |  |  |  |
| Appearance: Unfomfortable about appearance | 1.63*** | 1.55*** | 1.81*** | 1.55*** | 1.48*** | 1.70*** |
|  | (1.49 - 1.79) | (1.36 - 1.76) | (1.58 - 2.09) | (1.41 - 1.71) | (1.30 - 1.69) | (1.45 - 1.98) |
|  |  |  |  |  |  |  |
|  |  |  |  |  |  |  |
| Potential confounders | ✓ | ✓ | ✓ | ✓ | ✓ | ✓ |
|  |  |  |  |  |  |  |
| Observations | 3,075 | 1,570 | 1,502 | 3,075 | 1,570 | 1,499 |
| Pseudo-R² | .25 | .27 | .24 | .23 | .22 | .26 |

Odds ratios are displayed; 95% CI in parentheses; *** p<0.001, ** p<0.01, * p<0.05, + p<0.10; Potential confounders include sex (as appropriate), age, family status, educational level, occupational status, smoking status, alcohol intake, sports activities, vaccinated against Covid-19, presence of chronic diseases and self-rated health

Supplementary Table 8. Determinants of probable depression and probable anxiety. Results of multiple logistic regressions

| Independent variables | Probable depression  - Total sample | Probable depression  - Women | Probable depression  - Men | Probable anxiety -  Total sample | Probable anxiety  - Women | Probable anxiety  - Men |
| --- | --- | --- | --- | --- | --- | --- |
|  |  |  |  |  |  |  |
| Psychosocial impact: Difficulty doing your usual jobs | 2.11*** | 1.95*** | 2.32*** | 1.84*** | 1.59*** | 2.11*** |
|  | (1. ( - 2.40) | (1.60 - 2.37) | (1.94 - 2.78) | (1.61 - 2.10) | (1.31 - 1.93) | (1.75 - 2.55) |
|  |  |  |  |  |  |  |
| Potential confounders | ✓ | ✓ | ✓ | ✓ | ✓ | ✓ |
|  |  |  |  |  |  |  |
| Observations | 3,075 | 1,570 | 1,502 | 3,075 | 1,570 | 1,499 |
| Pseudo-R² | .26 | .27 | .26 | .23 | .21 | .27 |

Odds ratios are displayed; 95% CI in parentheses; *** p<0.001, ** p<0.01, * p<0.05, + p<0.10; Potential confounders include sex (as appropriate), age, family status, educational level, occupational status, smoking status, alcohol intake, sports activities, vaccinated against Covid-19, presence of chronic diseases and self-rated health
